# Supplementary material for: Correlations of intracranial pathology and cause of head injury with retinal hemorrhage in infants and toddlers: A multicenter, retrospective study by the J-HITs (Japanese Head injury of Infants and Toddlers study) group
Source: PLoS One. 2023 Mar 17;18(3):e0283297. doi: 10.1371/journal.pone.0283297 (PMC10022784; doi:10.1371/journal.pone.0283297)
Supplement: S1 Appendix — (DOCX) [file pone.0283297.s001.docx]

| age (months) | over 6 mos | sex | subdural hematoma | retinal hemorrhage | brain edema | skull fracture | contusion | epidural hematoma | subarachnoid hemorrhage | epilepsy/seizure | noticification to child guidance center | taken into custody | prosecuted as a criminal case | cause of injury explained by caregivers | categorized as nonaccidental trauma in this study |
| --- | --- | --- | --- | --- | --- | --- | --- | --- | --- | --- | --- | --- | --- | --- | --- |
| 1 | no | male | no | yes | yes | yes | no | no | yes | yes | yes | yes | yes | Confessed abuse | yes |
| 4 | no | male | yes | yes | no | yes | no | no | yes | yes | yes | yes | yes | Confessed abuse | yes |
| 2 | no | female | yes | yes | yes | no | no | no | yes | yes | yes | yes | yes | Unexplained convulsions | yes |
| 4 | no | male | yes | yes | no | no | yes | no | no | yes | yes | yes | yes | Unexplained convulsions | yes |
| 3 | no | male | yes | yes | no | no | yes | no | no | yes | yes | yes | yes | Confessed abuse | yes |
| 5 | no | female | yes | no | no | no | yes | no | no | yes | yes | yes | yes | Confessed abuse | yes |
| 2 | no | male | yes | no | yes | yes | no | no | no | yes | yes | yes | yes | Unexplained convulsions | yes |
| 2 | no | female | yes | yes | no | yes | no | no | no | yes | yes | yes | yes | Unexplained convulsions | yes |
| 4 | no | female | yes | yes | yes | no | no | no | no | yes | yes | yes | yes | Confessed abuse | yes |
| 4 | no | male | yes | yes | no | no | no | no | no | yes | yes | yes | yes | Confessed abuse | yes |
| 6 | yes | female | yes | yes | no | no | no | no | no | yes | yes | yes | yes | Unexplained convulsions | yes |
| 1 | no | male | yes | yes | no | yes | no | no | yes | no | yes | yes | yes | Unexplained coma | yes |
| 2 | no | male | yes | yes | no | no | no | no | no | no | yes | yes | yes | Unexplained coma | yes |
| 8 | yes | male | yes | yes | no | no | no | no | no | no | yes | yes | yes | Unexplained coma | yes |
| 20 | yes | male | yes | yes | yes | no | no | no | no | yes | yes | no | yes | Confessed abuse | yes |
| 11 | yes | female | no | no | no | yes | no | no | no | yes | yes | no | yes | Self-inflicted fall | no |
| 5 | no | male | yes | yes | no | no | no | no | no | yes | yes | yes | no | Other head injury | yes |
| 9 | yes | male | yes | yes | no | no | no | no | no | yes | yes | yes | no | Self-inflicted fall | yes |
| 7 | yes | male | no | no | no | yes | no | yes | no | no | yes | yes | unknown | Self-inflicted fall | yes |
| 4 | no | male | yes | yes | no | yes | yes | no | no | no | yes | yes | no | other unexplained event | yes |
| 1 | no | female | yes | yes | no | yes | no | no | yes | yes | yes | yes | unknown | Unexplained convulsions | yes |
| 3 | no | male | yes | yes | no | no | no | no | yes | yes | yes | yes | unknown | Unexplained convulsions | yes |
| 1 | no | male | yes | no | no | no | yes | yes | no | yes | yes | yes | unknown | Unexplained convulsions | yes |
| 1 | no | male | yes | yes | yes | yes | yes | no | no | yes | yes | yes | unknown | Unexplained convulsions | yes |
| 2 | no | male | yes | yes | yes | yes | no | no | no | yes | yes | yes | unknown | Unexplained convulsions | yes |
| 15 | yes | male | yes | yes | yes | no | no | no | no | yes | yes | yes | unknown | Self-inflicted fall | yes |
| 4 | no | male | yes | yes | no | no | no | no | no | yes | yes | yes | unknown | Unexplained convulsions | yes |
| 7 | yes | female | yes | yes | no | no | no | no | no | yes | yes | yes | unknown | Unexplained convulsions | yes |
| 3 | no | male | yes | no | no | no | no | no | no | yes | yes | yes | unknown | Confessed abuse | yes |
| 4 | no | male | yes | yes | no | yes | no | no | yes | no | yes | yes | unknown | Falling while being held by a parent | yes |
| 2 | no | male | yes | yes | no | yes | no | no | yes | no | yes | yes | unknown | Other head injury | yes |
| 3 | no | male | yes | no | no | yes | no | no | no | no | yes | yes | unknown | Confessed abuse | yes |
| 34 | yes | male | yes | yes | yes | no | no | no | no | no | yes | yes | unknown | Unexplained coma | yes |
| 4 | no | male | yes | no | yes | no | no | no | no | no | yes | yes | unknown | Confessed abuse | yes |
| 30 | yes | female | yes | yes | yes | no | no | no | no | no | yes | dead | unknown | Confessed abuse | yes |
| 4 | no | male | yes | yes | yes | yes | no | no | no | yes | yes | no | unknown | Other falls from <2m | no |
| 8 | yes | female | no | no | no | yes | no | no | yes | yes | yes | yes | no | Falling while being held by a parent | yes |
| 3 | no | female | yes | yes | no | no | no | no | yes | yes | yes | yes | no | Parents dropped | yes |
| 2 | no | female | yes | no | no | no | no | no | yes | yes | yes | yes | no | Unexplained convulsions | yes |
| 4 | no | male | yes | yes | no | yes | no | no | no | yes | yes | yes | no | Falling while being held by a parent | yes |
| 8 | yes | female | yes | yes | yes | no | no | no | no | yes | yes | yes | no | Confessed abuse | yes |
| 2 | no | female | yes | yes | no | no | no | no | no | yes | yes | yes | no | Unexplained convulsions | yes |
| 4 | no | female | yes | yes | no | no | no | no | no | yes | yes | yes | no | Unexplained convulsions | yes |
| 2 | no | male | yes | yes | no | no | no | no | no | yes | yes | yes | no | Unexplained convulsions | yes |
| 7 | yes | female | yes | yes | no | no | no | no | no | yes | yes | yes | no | Falling while being held by a parent | yes |
| 9 | yes | male | yes | yes | no | no | no | no | no | yes | yes | yes | no | Self-inflicted fall | yes |
| 6 | yes | male | yes | yes | no | no | no | no | no | yes | yes | yes | no | Unexplained convulsions | yes |
| 6 | yes | female | yes | no | no | no | no | no | no | yes | yes | yes | no | Unexplained convulsions | yes |
| 1 | no | male | no | no | no | yes | no | yes | no | no | yes | yes | no | Other head injury | yes |
| 9 | yes | male | no | no | no | yes | no | yes | no | no | yes | yes | no | Falling while being held by a parent | yes |
| 1 | no | male | no | yes | no | no | no | yes | no | no | yes | yes | no | Confessed abuse | yes |
| 11 | yes | female | no | no | no | yes | no | no | no | no | yes | yes | no | Falls from >2m | yes |
| 1 | no | male | yes | yes | yes | no | no | no | no | no | yes | yes | no | Unexplained coma | yes |
| 10 | yes | male | yes | yes | yes | no | no | no | no | no | yes | yes | no | Falling from a bed or sofa | yes |
| 5 | no | female | yes | yes | no | no | no | no | no | no | yes | yes | no | Falling while being held by a parent | yes |
| 3 | no | male | yes | yes | no | no | no | no | no | no | yes | yes | no | other unexplained event | yes |
| 9 | yes | male | yes | yes | no | no | no | no | no | no | yes | yes | no | Self-inflicted fall | yes |
| 9 | yes | male | yes | yes | no | no | no | no | no | no | yes | yes | no | Self-inflicted fall | yes |
| 8 | yes | male | yes | yes | no | no | no | no | no | no | yes | yes | no | Unexplained coma | yes |
| 2 | no | male | yes | yes | yes | no | no | no | no | yes | yes | no | no | Confessed abuse | yes |
| 4 | no | male | yes | yes | no | no | no | no | yes | yes | yes | no | no | Other falls from <2m | no |
| 8 | yes | male | yes | yes | yes | no | no | no | no | yes | yes | no | no | Self-inflicted fall | no |
| 20 | yes | male | yes | yes | yes | no | no | no | no | yes | yes | no | no | Falling from a bed or sofa | no |
| 9 | yes | female | yes | yes | no | no | no | no | no | yes | yes | no | no | Self-inflicted fall | no |
| 7 | yes | female | yes | yes | no | no | no | no | no | yes | yes | no | no | Unexplained convulsions | no |
| 9 | yes | male | yes | yes | no | no | no | no | no | yes | yes | no | no | Other falls from <2m | no |
| 8 | yes | male | yes | yes | no | no | no | no | no | yes | yes | no | no | Self-inflicted fall | no |
| 8 | yes | male | yes | yes | no | no | no | no | no | yes | yes | no | no | Self-inflicted fall | no |
| 10 | yes | male | yes | yes | no | no | no | no | no | yes | yes | no | no | Self-inflicted fall | no |
| 10 | yes | male | yes | yes | no | no | no | no | no | yes | yes | no | no | Self-inflicted fall | no |
| 12 | yes | male | yes | yes | no | no | no | no | no | yes | yes | no | no | Falling from a bed or sofa | no |
| 15 | yes | male | yes | no | no | no | no | no | no | yes | yes | no | no | Other head injury | no |
| 6 | yes | female | no | no | no | yes | no | yes | no | no | yes | no | no | Parents dropped | no |
| 9 | yes | female | no | no | no | yes | no | yes | no | no | yes | no | no | Falling from a bed or sofa | no |
| 41 | yes | male | yes | yes | yes | yes | no | no | no | no | yes | no | no | Falls from >2m | no |
| 2 | no | female | yes | yes | no | yes | no | no | no | no | yes | no | no | Falling while being held by a parent | no |
| 12 | yes | female | yes | yes | no | yes | no | no | no | no | yes | no | no | Self-inflicted fall | no |
| 0 | no | female | yes | yes | yes | no | no | no | no | no | yes | no | no | Other falls from <2m | no |
| 8 | yes | male | yes | yes | yes | no | no | no | no | no | yes | no | no | Falls from >2m | no |
| 10 | yes | male | yes | yes | yes | no | no | no | no | no | yes | no | no | Self-inflicted fall | no |
| 8 | yes | male | yes | yes | yes | no | no | no | no | no | yes | no | no | Falling from a bed or sofa | no |
| 3 | no | male | yes | yes | no | no | no | no | no | no | yes | no | no | Unexplained convulsions | no |
| 7 | yes | male | yes | yes | no | no | no | no | no | no | yes | no | no | Self-inflicted fall | no |
| 10 | yes | male | yes | yes | no | no | no | no | no | no | yes | no | no | Self-inflicted fall | no |
| 8 | yes | male | yes | yes | no | no | no | no | no | no | yes | no | no | Other head injury | no |
| 15 | yes | male | yes | yes | no | no | no | no | no | no | yes | no | no | Other head injury | no |
| 9 | yes | male | yes | no | no | no | no | no | no | no | yes | no | no | Falling from a bed or sofa | no |
| 45 | yes | male | no | no | no | yes | no | yes | no | yes | no | no | no | Falls from >2m | no |
| 2 | no | male | yes | no | no | yes | no | no | yes | yes | no | no | no | Falling while being held by a parent | no |
| 4 | no | male | yes | yes | yes | no | yes | no | no | yes | no | no | no | Falling while being held by a parent | no |
| 4 | no | male | yes | yes | yes | yes | no | no | no | yes | no | no | no | Motor vehicle accidents | no |
| 10 | yes | male | yes | no | no | yes | no | no | no | yes | no | no | no | Self-inflicted fall | no |
| 11 | yes | male | yes | no | yes | no | no | no | no | yes | no | no | no | Falling from a bed or sofa | no |
| 4 | no | male | yes | yes | no | no | no | no | no | yes | no | no | no | Motor vehicle accidents | no |
| 13 | yes | female | yes | yes | no | no | no | no | no | yes | no | no | no | Falling from a bed or sofa | no |
| 14 | yes | male | yes | yes | no | no | no | no | no | yes | no | no | no | Other falls from <2m | no |
| 9 | yes | male | yes | yes | no | no | no | no | no | yes | no | no | no | Self-inflicted fall | no |
| 11 | yes | male | yes | yes | no | no | no | no | no | yes | no | no | no | Self-inflicted fall | no |
| 7 | yes | male | yes | yes | no | no | no | no | no | yes | no | no | no | Falling from a bed or sofa | no |
| 16 | yes | male | yes | yes | no | no | no | no | no | yes | no | no | no | Falling from a bed or sofa | no |
| 1 | no | female | yes | no | no | no | no | no | no | yes | no | no | no | Parents dropped | no |
| 1 | no | male | yes | no | no | no | no | no | no | yes | no | no | no | Unexplained convulsions | no |
| 12 | yes | male | yes | no | no | no | no | no | no | yes | no | no | no | Self-inflicted fall | no |
| 11 | yes | male | yes | no | no | no | no | no | no | yes | no | no | no | Self-inflicted fall | no |
| 8 | yes | male | yes | no | no | no | no | no | no | yes | no | no | no | Falling from a bed or sofa | no |
| 7 | yes | male | yes | no | no | no | no | no | no | yes | no | no | no | Falling from a bed or sofa | no |
| 7 | yes | male | yes | no | no | no | no | no | no | yes | no | no | no | Falling from a bed or sofa | no |
| 1 | no | male | no | no | no | yes | yes | no | yes | no | no | no | no | Parents dropped | no |
| 4 | no | female | no | no | no | no | yes | no | yes | no | no | no | no | Parents dropped | no |
| 7 | yes | male | yes | no | no | yes | no | no | yes | no | no | no | no | Parents dropped | no |
| 0 | no | female | no | no | no | yes | no | no | yes | no | no | no | no | Parents dropped | no |
| 5 | no | female | no | no | no | yes | no | no | yes | no | no | no | no | Parents dropped | no |
| 2 | no | male | no | no | no | yes | no | no | yes | no | no | no | no | Falling while being held by a parent | no |
| 3 | no | male | no | no | no | yes | no | no | yes | no | no | no | no | Falling while being held by a parent | no |
| 2 | no | male | no | no | no | yes | no | no | yes | no | no | no | no | Parents dropped | no |
| 3 | no | male | no | no | no | yes | no | no | yes | no | no | no | no | Parents dropped | no |
| 1 | no | male | no | no | no | yes | no | no | yes | no | no | no | no | Falling from a bed or sofa | no |
| 10 | yes | female | no | no | no | yes | no | no | yes | no | no | no | no | Bicycle accidents | no |
| 48 | yes | male | no | no | no | yes | no | no | yes | no | no | no | no | Motor vehicle accidents | no |
| 1 | no | female | yes | no | no | no | no | no | yes | no | no | no | no | Parents dropped | no |
| 3 | no | female | no | no | no | no | no | no | yes | no | no | no | no | Other falls from <2m | no |
| 5 | no | female | yes | no | no | yes | yes | yes | no | no | no | no | no | Parents dropped | no |
| 48 | yes | female | yes | no | no | yes | yes | yes | no | no | no | no | no | Other falls from <2m | no |
| 8 | yes | male | no | no | no | yes | yes | yes | no | no | no | no | no | Other falls from <2m | no |
| 4 | no | female | yes | no | no | yes | no | yes | no | no | no | no | no | Parents dropped | no |
| 3 | no | male | yes | no | no | yes | no | yes | no | no | no | no | no | Falling while being held by a parent | no |
| 1 | no | female | no | no | no | yes | no | yes | no | no | no | no | no | Falling while being held by a parent | no |
| 0 | no | female | no | no | no | yes | no | yes | no | no | no | no | no | Parents dropped | no |
| 0 | no | female | no | no | no | yes | no | yes | no | no | no | no | no | Parents dropped | no |
| 1 | no | female | no | no | no | yes | no | yes | no | no | no | no | no | Parents dropped | no |
| 1 | no | female | no | no | no | yes | no | yes | no | no | no | no | no | Parents dropped | no |
| 1 | no | female | no | no | no | yes | no | yes | no | no | no | no | no | Parents dropped | no |
| 4 | no | female | no | no | no | yes | no | yes | no | no | no | no | no | Parents dropped | no |
| 1 | no | male | no | no | no | yes | no | yes | no | no | no | no | no | Falling while being held by a parent | no |
| 4 | no | male | no | no | no | yes | no | yes | no | no | no | no | no | Falling while being held by a parent | no |
| 2 | no | male | no | no | no | yes | no | yes | no | no | no | no | no | Parents dropped | no |
| 5 | no | male | no | no | no | yes | no | yes | no | no | no | no | no | Other falls from <2m | no |
| 0 | no | male | no | no | no | yes | no | yes | no | no | no | no | no | Other head injury | no |
| 6 | yes | female | no | no | no | yes | no | yes | no | no | no | no | no | Other falls from <2m | no |
| 6 | yes | female | no | no | no | yes | no | yes | no | no | no | no | no | Other falls from <2m | no |
| 13 | yes | female | no | no | no | yes | no | yes | no | no | no | no | no | Other falls from <2m | no |
| 15 | yes | female | no | no | no | yes | no | yes | no | no | no | no | no | Other falls from <2m | no |
| 19 | yes | female | no | no | no | yes | no | yes | no | no | no | no | no | Other falls from <2m | no |
| 43 | yes | female | no | no | no | yes | no | yes | no | no | no | no | no | Other falls from <2m | no |
| 19 | yes | female | no | no | no | yes | no | yes | no | no | no | no | no | Falling from a bed or sofa | no |
| 8 | yes | female | no | no | no | yes | no | yes | no | no | no | no | no | other unexplained event | no |
| 8 | yes | male | no | no | no | yes | no | yes | no | no | no | no | no | Bicycle accidents | no |
| 6 | yes | male | no | no | no | yes | no | yes | no | no | no | no | no | Parents dropped | no |
| 14 | yes | male | no | no | no | yes | no | yes | no | no | no | no | no | Parents dropped | no |
| 6 | yes | male | no | no | no | yes | no | yes | no | no | no | no | no | Other falls from <2m | no |
| 7 | yes | male | no | no | no | yes | no | yes | no | no | no | no | no | Other falls from <2m | no |
| 9 | yes | male | no | no | no | yes | no | yes | no | no | no | no | no | Other falls from <2m | no |
| 29 | yes | male | no | no | no | yes | no | yes | no | no | no | no | no | Other falls from <2m | no |
| 6 | yes | male | no | no | no | yes | no | yes | no | no | no | no | no | Falling from a bed or sofa | no |
| 7 | yes | male | no | no | no | yes | no | yes | no | no | no | no | no | Falling from a bed or sofa | no |
| 8 | yes | male | no | no | no | no | no | yes | no | no | no | no | no | Falls from >2m | no |
| 16 | yes | male | no | no | no | no | no | yes | no | no | no | no | no | Other falls from <2m | no |
| 1 | no | male | yes | no | no | yes | yes | no | no | no | no | no | no | Parents dropped | no |
| 2 | no | male | yes | no | no | yes | yes | no | no | no | no | no | no | Parents dropped | no |
| 3 | no | male | no | no | no | yes | yes | no | no | no | no | no | no | Parents dropped | no |
| 31 | yes | male | no | no | no | yes | yes | no | no | not described | no | no | no | Falls from >2m | no |
| 6 | yes | male | no | no | no | yes | yes | no | no | no | no | no | no | Falling while being held by a parent | no |
| 2 | no | female | yes | no | no | no | yes | no | no | no | no | no | no | Parents dropped | no |
| 7 | yes | male | no | no | no | no | yes | no | no | no | no | no | no | Falling while being held by a parent | no |
| 1 | no | female | no | no | no | yes | no | no | no | no | no | no | no | Parents dropped | no |
| 3 | no | female | no | no | no | yes | no | no | no | no | no | no | no | Parents dropped | no |
| 3 | no | female | no | no | no | yes | no | no | no | no | no | no | no | Parents dropped | no |
| 3 | no | female | no | no | no | yes | no | no | no | no | no | no | no | Parents dropped | no |
| 4 | no | female | no | no | no | yes | no | no | no | no | no | no | no | Parents dropped | no |
| 4 | no | female | no | no | no | yes | no | no | no | no | no | no | no | Parents dropped | no |
| 1 | no | female | no | no | no | yes | no | no | no | no | no | no | no | Other falls from <2m | no |
| 4 | no | female | no | no | no | yes | no | no | no | no | no | no | no | Falling while being held by a parent | no |
| 2 | no | male | no | no | no | yes | no | no | no | no | no | no | no | Falling while being held by a parent | no |
| 4 | no | male | no | no | no | yes | no | no | no | no | no | no | no | Falling while being held by a parent | no |
| 5 | no | male | no | no | no | yes | no | no | no | no | no | no | no | Falling while being held by a parent | no |
| 5 | no | male | no | no | no | yes | no | no | no | no | no | no | no | Falling while being held by a parent | no |
| 2 | no | male | no | no | no | yes | no | no | no | no | no | no | no | Falling while being held by a parent | no |
| 0 | no | male | no | no | no | yes | no | no | no | no | no | no | no | Parents dropped | no |
| 1 | no | male | no | no | no | yes | no | no | no | no | no | no | no | Parents dropped | no |
| 3 | no | male | no | no | no | yes | no | no | no | no | no | no | no | Parents dropped | no |
| 3 | no | male | no | no | no | yes | no | no | no | no | no | no | no | Parents dropped | no |
| 3 | no | male | no | no | no | yes | no | no | no | no | no | no | no | Parents dropped | no |
| 5 | no | male | no | no | no | yes | no | no | no | no | no | no | no | Parents dropped | no |
| 5 | no | male | no | no | no | yes | no | no | no | no | no | no | no | Parents dropped | no |
| 4 | no | male | no | no | no | yes | no | no | no | no | no | no | no | Other falls from <2m | no |
| 5 | no | male | no | no | no | yes | no | no | no | no | no | no | no | Self-inflicted fall | no |
| 4 | no | male | no | no | no | yes | no | no | no | no | no | no | no | Falling from a bed or sofa | no |
| 5 | no | male | no | no | no | yes | no | no | no | no | no | no | no | other unexplained event | no |
| 13 | yes | female | no | no | no | yes | no | no | no | no | no | no | no | Motor vehicle accidents | no |
| 23 | yes | female | no | no | no | yes | no | no | no | no | no | no | no | Bicycle accidents | no |
| 13 | yes | female | no | no | no | yes | no | no | no | no | no | no | no | Falls from >2m | no |
| 19 | yes | female | no | no | no | yes | no | no | no | no | no | no | no | Falling while being held by a parent | no |
| 6 | yes | female | no | no | no | yes | no | no | no | no | no | no | no | Parents dropped | no |
| 22 | yes | female | no | no | no | yes | no | no | no | no | no | no | no | Parents dropped | no |
| 6 | yes | female | no | no | no | yes | no | no | no | no | no | no | no | Other falls from <2m | no |
| 8 | yes | female | no | no | no | yes | no | no | no | no | no | no | no | Other falls from <2m | no |
| 11 | yes | female | no | no | no | yes | no | no | no | no | no | no | no | Other falls from <2m | no |
| 12 | yes | female | no | no | no | yes | no | no | no | no | no | no | no | Other falls from <2m | no |
| 13 | yes | female | no | no | no | yes | no | no | no | no | no | no | no | Other falls from <2m | no |
| 11 | yes | female | no | no | no | yes | no | no | no | no | no | no | no | other unexplained event | no |
| 36 | yes | female | no | no | no | yes | no | no | no | no | no | no | no | Motor vehicle accidents | no |
| 8 | yes | female | no | no | no | yes | no | no | no | no | no | no | no | Parents dropped | no |
| 20 | yes | male | no | no | no | yes | no | no | no | no | no | no | no | Bicycle accidents | no |
| 20 | yes | male | no | no | no | yes | no | no | no | no | no | no | no | Falling while being held by a parent | no |
| 8 | yes | male | no | no | no | yes | no | no | no | no | no | no | no | Parents dropped | no |
| 8 | yes | male | no | no | no | yes | no | no | no | no | no | no | no | Parents dropped | no |
| 11 | yes | male | no | no | no | yes | no | no | no | no | no | no | no | Other falls from <2m | no |
| 15 | yes | male | no | no | no | yes | no | no | no | no | no | no | no | Other falls from <2m | no |
| 15 | yes | male | no | no | no | yes | no | no | no | no | no | no | no | Other falls from <2m | no |
| 20 | yes | male | no | no | no | yes | no | no | no | no | no | no | no | Other falls from <2m | no |
| 8 | yes | male | no | no | no | yes | no | no | no | no | no | no | no | Self-inflicted fall | no |
| 9 | yes | male | no | no | no | yes | no | no | no | no | no | no | no | Self-inflicted fall | no |
| 12 | yes | male | no | no | no | yes | no | no | no | no | no | no | no | Self-inflicted fall | no |
| 14 | yes | male | no | no | no | yes | no | no | no | no | no | no | no | Self-inflicted fall | no |
| 7 | yes | male | no | no | no | yes | no | no | no | no | no | no | no | Falling from a bed or sofa | no |
| 7 | yes | male | no | no | no | yes | no | no | no | no | no | no | no | Falling from a bed or sofa | no |
| 6 | yes | male | no | no | no | yes | no | no | no | no | no | no | no | other unexplained event | no |
| 0 | no | female | yes | yes | yes | no | no | no | no | no | no | no | no | Birth injury | no |
| 0 | no | male | yes | no | yes | no | no | no | no | no | no | no | no | Birth injury | no |
| 9 | yes | female | yes | yes | no | no | no | no | no | no | no | no | no | Self-inflicted fall | no |
| 6 | yes | female | yes | yes | no | no | no | no | no | no | no | no | no | Self-inflicted fall | no |
| 8 | yes | male | yes | yes | no | no | no | no | no | no | no | no | no | Self-inflicted fall | no |
| 13 | yes | male | yes | yes | no | no | no | no | no | no | no | no | no | Self-inflicted fall | no |
| 16 | yes | male | yes | yes | no | no | no | no | no | no | no | no | no | Falling from a bed or sofa | no |
| 19 | yes | male | yes | yes | no | no | no | no | no | no | no | no | no | Other head injury | no |
| 2 | no | female | yes | no | no | no | no | no | no | no | no | no | no | Motor vehicle accidents | no |
| 4 | no | female | yes | no | no | no | no | no | no | no | no | no | no | Parents dropped | no |
| 0 | no | male | yes | no | no | no | no | no | no | no | no | no | no | Birth injury | no |
| 48 | yes | female | yes | no | no | no | no | no | no | no | no | no | no | Other falls from <2m | no |
| 8 | yes | female | yes | no | no | no | no | no | no | no | no | no | no | Falling from a bed or sofa | no |
| 9 | yes | female | yes | no | no | no | no | no | no | no | no | no | no | Falling from a bed or sofa | no |
| 17 | yes | male | yes | no | no | no | no | no | no | no | no | no | no | Bicycle accidents | no |
| 10 | yes | male | yes | no | no | no | no | no | no | no | no | no | no | Other falls from <2m | no |
| 18 | yes | male | yes | no | no | no | no | no | no | no | no | no | no | Other falls from <2m | no |
| 10 | yes | male | yes | no | no | no | no | no | no | no | no | no | no | Other falls from <2m | no |
| 10 | yes | male | yes | no | no | no | no | no | no | no | no | no | no | Self-inflicted fall | no |
| 10 | yes | male | yes | no | no | no | no | no | no | no | no | no | no | Falling from a bed or sofa | no |
| 13 | yes | male | yes | no | no | no | no | no | no | no | no | no | no | Falling from a bed or sofa | no |
| 12 | yes | male | yes | no | no | no | no | no | no | no | no | no | no | Other head injury | no |
