# Supplementary material for: Correlations of intracranial pathology and cause of head injury with retinal hemorrhage in infants and toddlers: A multicenter, retrospective study by the J-HITs (Japanese Head injury of Infants and Toddlers study) group
Source: PLoS One. 2023 Mar 17;18(3):e0283297. doi: 10.1371/journal.pone.0283297 (PMC10022784; doi:10.1371/journal.pone.0283297)
Supplement: Attachment — Submitted filename: Response to reviewers.docx [file pone.0283297.s002.docx]

Response to Reviewers

When submitting your revision, we need you to address these additional requirements.

1. Please ensure that your manuscript meets PLOS ONE's style requirements, including those for file naming. The PLOS ONE style templates can be found at 
<https://journals.plos.org/plosone/s/file?id=wjVg/PLOSOne_formatting_sample_main_body.pdf> and 
<https://journals.plos.org/plosone/s/file?id=ba62/PLOSOne_formatting_sample_title_authors_affiliations.pdf>

2. Thank you for stating the following in the Competing Interests section: 
"Masahiro Nonaka and Young-Soo Park have written statements and appeared in court in child abuse cases both on the request of the prosecutor and the defense. Atsuko Harada has written statements and appeared in court in child abuse cases on the request of the prosecutor. This does not alter our adherence to PLOS ONE policies on sharing data and materials."

Please confirm that this does not alter your adherence to all PLOS ONE policies on sharing data and materials, by including the following statement: "This does not alter our adherence to  PLOS ONE policies on sharing data and materials.” (as detailed online in our guide for authors <http://journals.plos.org/plosone/s/competing-interests>).  If there are restrictions on sharing of data and/or materials, please state these. Please note that we cannot proceed with consideration of your article until this information has been declared.

Please include your updated Competing Interests statement in your cover letter; we will change the online submission form on your behalf.

**Author’s Response:** There are no particular changes to be made. All data used in this study are disclosed as supplementary data in an anonymized form.

3. Please include your full ethics statement in the ‘Methods’ section of your manuscript file. In your statement, please include the full name of the IRB or ethics committee who approved or waived your study, as well as whether or not you obtained informed written or verbal consent. If consent was waived for your study, please include this information in your statement as well.

**Author’s Response:** The following sentences were added.

Change to Text: Page 5, Lines 6-10

IRB/ethics committee approval and a statement regarding patient consent

The protocol for this study was approved by the Ethics Committee of Kansai Medical University (No. 2019232). Need for written patient consent was waived by the ethics committee because data were deidentified. Institutional review board approval was obtained from all participants’ institutions prior to submitting cases for this study.

1. Is the manuscript technically sound, and do the data support the conclusions?

The manuscript must describe a technically sound piece of scientific research with data that supports the conclusions. Experiments must have been conducted rigorously, with appropriate controls, replication, and sample sizes. The conclusions must be drawn appropriately based on the data presented.

Reviewer #1: Partly

Reviewer #2: Yes

2. Has the statistical analysis been performed appropriately and rigorously?

Reviewer #1: Yes

Reviewer #2: Yes

3. Have the authors made all data underlying the findings in their manuscript fully available?

The [PLOS Data policy](http://www.plosone.org/static/policies.action#sharing) requires authors to make all data underlying the findings described in their manuscript fully available without restriction, with rare exception (please refer to the Data Availability Statement in the manuscript PDF file). The data should be provided as part of the manuscript or its supporting information, or deposited to a public repository. For example, in addition to summary statistics, the data points behind means, medians and variance measures should be available. If there are restrictions on publicly sharing data—e.g. participant privacy or use of data from a third party—those must be specified.

Reviewer #1: No

Reviewer #2: Yes

**Author’s Response:** Please note that all data used in this study are disclosed as supplementary data in an anonymized form.

- 4. Is the manuscript presented in an intelligible fashion and written in standard English?

  PLOS ONE does not copyedit accepted manuscripts, so the language in submitted articles must be clear, correct, and unambiguous. Any typographical or grammatical errors should be corrected at revision, so please note any specific errors here.

Reviewer #1: No

Reviewer #2: Yes

5. Review Comments to the Author

Please use the space provided to explain your answers to the questions above. You may also include additional comments for the author, including concerns about dual publication, research ethics, or publication ethics. (Please upload your review as an attachment if it exceeds 20,000 characters)

Reviewer #1: The goals of this study are appropriate, but much needs clarification. Some of their reference literature is of very poor quality and some significant papers are missed.
1) I will just speak to the body of the manuscript, but that implies also a need to revise the abstract.

Author’s response: The abstract as well as the body of the manuscript has been changed.

2) Methods, p3,L 15_ what is considered head trauma. This threshold for inclusion needs to be defined.

Author’s response: We defined head trauma in this paper as follows

Change to text: Page 3, Lines 13-16

The definition of head trauma in our study included cases in which the parent or guardian stated during the interview that the child had suffered a head injury, as well as cases in which there were findings on imaging that were thought to be due to head trauma.　Furthermore, Patients with imaging findings, such as skull fractures or intracranial injuries, were included. Patients with no imaging findings, patients with only extracranial findings, and patients with no apparent findings on imaging were excluded.

3) P4, L6-8 It's good to document both the caretaker's explanation and the cPT diagnosis.

Author’s response: A new Table 3 summarizes the injury mechanism described by each guardian and which factors tend to be judged as abuse.

Change to text: Page 7, Lines 6-11

Table 3 shows the injury mechanisms described by the parents in our study and the percentage of nonaccidental judgments for each. The presence or absence of retinal hemorrhages is also noted. The cases in which the parents did not fully explain the cause of the injury tended to be judged as abuse, while the cases in which the cause of the injury was fully explained tended to be judged as accidental. Retinal hemorrhage was present in 65.6% of self-inflicted falls.

Page 8, Lines 1-3

Table 3.　Parents' description of the injury mechanism and determination of nonaccidental or accidental injury in this study.

4) P4, L13_ The authors need to provide information about the subjects excluded for lack of a retinal exam.

Author’s response:All 452 cases have already been reported. The present study is a detailed reanalysis of the presence/absence of retinal hemorrhage. References are listed below.

Change to text: Page 4, Line 12

[1]

5) P4, L17_it's OK to include confessions as Dx of abuse, but to require that the kids were placed in temporary custody is the CPT suspected abuse is incorrect-the legal system is more flawed in it's abuse assessment. I'd just go with the CPT opinion.

Author’s response: In Japan, placement of temporary custody is implemented only by the decision of the Child Guidance Center, not by a court decision. Furthermore, In Japan, the CPT's opinion is not reported to the patient's physician for privacy purposes. So the CPT's decision is known to physicians only when the patient is taken into custody.

6) P4, L24-"self-inflicted" needs more explanation and description.

Author’s response: We have explained about self-inflicted falls as follows

Change to text: Page 5, Lines 2-4

Self-inflicted falls occurs when an infant falls backward and bruises the back of the head, mainly when trying to stand while holding onto other objects.

7) The initial tables in results, beginning P5, L19-Although univariate ORs are later tabulated, it would help to see them in the initial tables.

Author’s response: We have also included ORs in Tables 1 and 2 in response to the reviewer’s suggestion.

Change to text: Please see new Table 1 and 2

8) Top table, P6-the authors need to state whether it is brain of scalp that was contused.

Author’s response: The word “brain” was inserted before “contusion” to clarify this point.

Change to text: Please see new Table 1, 3, and 4

9) P, 6, L 3-See Dr Morad's 2 papers from 2004 in JAAPOS & recent Simon paper JAAPOS 2022 doi: 10/1016.j.jaapos.2022.10.003

Author’s response: We have cited the references given and added the following text

Change to text: Page 11, Lines 12-15

There have been reports of shaken baby syndrome, in which retinal hemorrhage was observed despite the absence of intracranial findings {Morad, 2004 #170}{Morad, 2004 #171}{Simon, 2022 #175}, but in our study, only cases with some findings on CT or MRI were analyzed, so it is unclear to what extent retinal hemorrhage is observed in cases without intracranial findings.

10) P6, bottom table I note that with Major accidents: bicycle accidents, MVAs and falls > 2 M that far fewer kids have RH than children with Minor accidents: self-inflicted falls, bed or sofa falls and other falls < 2 M. This implies to me that the original data is incorrect. Most likely the children within the later groups were miss=diagnosed as not abuse. these are common false explanations.

Author’s response: Among the minor injuries in the current study, self-inflicted falls were significantly associated with retinal hemorrhage. In order to examine whether our data are accurate, we have analyzed the frequency and characteristics of self-inflicted falls from each participating facility in Table 5.

Change to text; Page 9, Lines 10-11, Page 10 , Lines 1-14 + Table 5

Self-inflicted falls, which were found to be significantly different in the multivariate analysis, have not been reported to be a factor associated with retinal hemorrhage. Therefore, we further examined the data in detail. Table 5 shows a detailed examination of the characteristics of self-inflicted falls by each facility. The results showed that self-inflicted falls were present in all facilities, ranging from 2.0-27.8% of the total number.

Self-inflicted falls were more common among boys in all facilities, and the average age ranged from 9 to 11 months. Parents/caregivers stated that 60-100% (mean 75.0%) of the injuries were caused by backward falls from a standing position, and 0-40% (mean 18.8%) were caused by backward falls from a sitting position. Retinal hemorrhages were present in 50-100% (mean 65.6%) and subdural hematomas in 58.3% to 100% (mean 78.1%). Skull fractures were observed in 0-50% of cases (mean 28.1%), and brain edemas were present in 0-16.7% (mean 9.4%, total 3 cases). There was only one case of subarachnoid hemorrhage and no cases of epidural hematoma or cerebral contusion.

Page 12, Lines 4-15

In our study, the cases of self-inflicted falls with abnormalities on CT or MRI were characterized by boys around 9-11 months of age, with backward falls as the cause of injury, accompanied by subdural hematoma and retinal hemorrhage, but without brain edema or brain contusion. Self-inflicted falls were also found at all of the facilities in the study, suggesting that there is unlikely to be a bias in favor of a particular facility. We considered that many of the cases were not determined to be nonaccidental because many of the patients had no parenchymal brain damage and had a good prognosis. We do not deny that this result may be due to the lack of strict evaluation of abuse in Japan as a whole. On the other hand, various reports from Japan pointed out that retinal hemorrhage and subdural hematoma occur in short fall, which is different from Western reports {Nonaka, 2022 #176}. The presence of these cases may be unique to Japan.

11) This might be an artifact of the authors failing to separate subjects into a few poster-pole, non-diagnostic RH vs many, to the periphery with or without schisis. This finding makes most of their conclusions suspect.

Author’s response: The intent of our study is to determine what factors influence retinal hemorrhage regardless of severity. So, we did not evaluate the severity of the retinal hemorrhage in this study. We added the following sentence in the limitation.

Change to text: Page 13, Lines 20-23 In addition, only the presence or absence of retinal hemorrhage was considered for this study. Whether retinal hemorrhage was unilateral or bilateral, or the severity of the hemorrhage was not considered. This needs to be addressed in future studies.

12) P9, L1-Though EDH is an impact injury and SAH can result from either impact or inertial injury. Kids with EDH can have inertial/rotational deceleration at impact. So it is unfair to conclude that impact injuries or "external forces" per se caused these injuries.

Author’s response: The following text has been deleted. “Still, some were associated with epidural hematoma or subarachnoid hemorrhage, so the possibility that an external force caused retinal hemorrhage to the head cannot be ruled out.”

Change to text: The deleted text was on Page 11, Line 10

13) P 9, L3-4- seems entirely unsupported by their data and of unclear meaning-it should be deleted.

Author’s response: The following sentence has been deleted. “However, it was unclear whether the force applied to the head needed to be as strong as an act of violence by the guardian to cause a retinal hemorrhage.”

Change to text: The deleted text was on Page 11, Line 10

14) P9, L12-15 Gardner's case is also very suspect. It doesn't warrant inclusion as evidence. But Atkinson's series is reputable.

Author’s response: We have removed the description of Gardner's paper. “Gardner reported a witnessed case of subdural hematoma with retinal hemorrhage due to a backward fall [9].”

Change to text: The deleted text was on Page 11, Line 23

15) Top P 10. Terson's is very rare in infants. L6-7-the retinal vein also has acessory outflow paths that prevent it from causing retinal hypertension if more proximal obstruction Occurs_See Morad Clin & Exp Ophth 2010;38:514. {Morad, 2010 #138}

Author’s response: We have removed the sentence about Terson. On the other hand, we described about birth-related retinal hemorrhage and the current possible mechanisms.

Change to text: Page 12 Line 24, Page 13, Lines 1-4

Birth-related retinal hemorrhage has been shown to occur frequently {Watts, 2013 #177}, especially in normal vaginal deliveries{Cho, 2021 #178} . It is hypothesized to occur when the infant head passes through a narrow parturient passage resulting in increased intracranial pressure, however this has not been proven{Cho, 2021 #178}.

16) P 10, L11_ Shiau also discussed ICP Arch peds Adol Med 2012;166:623 & Minns Devel Med Child Neurol 2017;59:597 found that although intracranial hemorrhages such as SDH commonly occur in accidental and abusive TBI that the presence of severe RH was not associated with the ICP but instead the initial trauma.

Author’s response: We have cited the references given and added the following text

Change to text: Page 12, Lines 21-24

Furthermore, several reports point out that in cases with severe retinal hemorrhage and intracranial hemorrhages such as subdural hematoma, retinal hemorrhage was not associated with the increased intracranial pressure but instead the initial trauma[19,12].

17) P10, L19_ Thiblin's study can be discounted. he's a lousy and dishonest researcher-Greeley Acta Paediatrica 2022 doi: 10.1111/apa.16488, {Greeley, 2022 #173} Stray-Pederson Acta Paediatrica 2022;11:793 {Stray-Pedersen, 2022 #174}

Author’s response: In accordance with the Reviewer's comments, the following text has been removed. :

“More recently, it has been reported that retinal hemorrhage may appear with a nonspecific cause associated with intracranial lesions such as subdural hematomas.”

”Thiblin et al. compared the medical findings and types of trauma reported in infants with and without retinal hemorrhage. They found that intracranial pathology was significantly more common in retinal hemorrhage cases (97%) than in non-retinal hemorrhage cases (13%).”

Change to text: The deleted text was on Page 3, Line 5, and Page 13, Line 8

18) P110. L 24- Saying that coats found iCP had a role in causing RH is incorrect, they only implicated indirect rotational acceleration forces. Theey didn't even look at ICP.

Author’s response: In response to the Reviewer's comments, we have revised the text as follows

Change to text: Page 13, Lines11-13

Coats et al. examined the characteristics of ocular hemorrhages caused by a single rapid head rotation in neonatal pigs [7]. They also examined intracranial findings and found subdural hematomas in 43 of the 46 cases. These results indicate that the force that produce subdural hematoma are similar to those that produce retinal hemorrhage.

19) P11, L8: Although some abuse cases might be over-diagnosed in this study, I remain overwhelmingly concerned that much abuse still has been missed. se my comments re the Table on P6.

Author’s response: We specifically reanalyzed self-inflicted falls. We found that many of the cases had no brain parenchymal damage and probably had a good prognosis, despite the presence of retinal hemorrhage and subdural hematoma. This may be the reason why many of the cases were judged to have been accidents. Please see my response in 10). We also have added the following text to the Study limitation.

Change to text: Page 13, Lines 18-20

Conversely, the high incidence of retinal hemorrhage due to minor head injury may indicate that nonaccidental cases is underestimated.

20) Although some abuse cases might be over-diagnosed in this study, I remain overwhelmingly concerned that much abuse still has been missed.

Author’s response: Please see my response in 10).

21) Above all I feel the authors are unjustified in their conclusions that RH result from the pressure from SDH and intra-cranial injuries as opposed to the same forces causing the SDH also causing the RH simultaneously and independently as a response to the forces experienced.

Author’s response: We have removed the sentence “These results indicate that retinal hemorrhage is more likely to occur in association with subdural hematoma than with direct injury” from the abstract and the conclusion.

Change to text: The deleted text was on Page 2. Line 15, and Page 14, Line 4

22) It's also problematic in this era to not separate minor and major RHs. Their specificity for abuse or major trauma is very different.

Author’s response: As the reviewer pointed out, the severity of retinal hemorrhage is very important to investigate the association with AHT.

However, we conducted this study because we believe that nothing can be done without first examining what factors cause retinal hemorrhage itself before examining the relationship with the severity of retinal hemorrhage.

Of course, based on our results, we think that in the future we need to conduct a multicenter study to examine the severity of retinal hemorrhage based on uniform criteria. We have added the following text to the Study limitation

Change to text: Page 13, Lines 20-23

In addition, only the presence or absence of retinal hemorrhage was considered, not whether it was unilateral or bilateral or the severity of the hemorrhage. This needs to be addressed in future studies.

Reviewer #2: Accept, however figures are not provided
Good English language , clear message delivered . The statistics are satisfactory. The topic is interesting and applicable

Author’s response: Thank you for the positive feedback and I will make sure to upload the Figures.
