# Supplementary material for: Correlations of intracranial pathology and cause of head injury with retinal hemorrhage in infants and toddlers: A multicenter, retrospective study by the J-HITs (Japanese Head injury of Infants and Toddlers study) group
Source: PLoS One. 2023 Mar 17;18(3):e0283297. doi: 10.1371/journal.pone.0283297 (PMC10022784; doi:10.1371/journal.pone.0283297)
Supplement: Attachment — Submitted filename: Response to reviewers R2.docx [file pone.0283297.s003.docx]

Response to Reviewers

Reviewer #1: The paper is greatly improved, but still needs some minor work, partly to educate non-Japanese audiences and partly interpretive.

**Author’s Response:** We thank the reviewer for valuable comments.

１）Page 3 or tracked changes document, line 21, I'd add:".....intracranial injuries of trauma who lacked caretaker histories of a traumatic event were included."

**Author’s Response:** The following sentences were added.

Change to text: Page 3, Lines 17-18

intracranial injuries of trauma who lacked caretaker histories of a traumatic event were included

2) Page 4, L10-you indicate caretaker history was entered as the injury cause of the "final injury mechanism". Do you mean historical or your final judgment?

**Author’s Response:** Since the cause of the injury described by the guardian may change, it means that the most recent description of how the injury occurred was recorded. To make this point clear, the sentence was rewritten.

Change to text: Page 4, Lines 9-10

If there were any changes in the parents’ explanation of the cause of the injury, they were noted verbatim, and the most recent description of the injury, as stated by the parents, was included in the study.

3) Page 4L13-115 and your response to reviewers point 5.: In that point you seem to suggest that if the Child Guidance Center is responsible for the decision whether to place the child in custody and only if this occurs is the referring providers notified of the abuse or not abuse judgment. I'm not sure I understand that correctly. But the text should document the actual practice. In you final diagnosis of abuse if fyou are your relying on caretaker history or the Child Guidance Center decision or something else. If the CGC, doesn't this imply you are naive to some decisions of whether abuse occurred or not. This whole section needs to be revised to educate the reader's about the Japanese process and what you are using when you say a child was abused or not.

**Author’s Response:** The following sentences were added.

Change to text: Page 5, Lines 1-12

The Childs Guidance Center in Japan receives notifications of suspected cases of abuse at medical institutions, etc., and determines whether or not abuse actually occurred. The CGC determines whether or not the child should be placed in temporary custody based on the child's history of visits to medical institutions and health checkups, whether or not the sibling has been abused, and the results of a diagnosis by a third-party doctor. The process and reasons for how the determination of whether or not abuse was made will not be communicated to the medical institution that notified of the abuse. In cases where abuse is strongly suspected, the child is almost always given temporary protection. On the other hand, cases that are determined to be unlikely to be abusive are not protected. Therefore, in this study, the cases that were temporarily protected by CGC were judged as abuse, and those that were not protected were judged as accidents.

5) In Table 2, P7: I remain concerned that self-inflicted falls, falling from a bed or sofa and other < 2 m falls seem to have more morbid consequences than falls fro greater heights, MVAs and bicycle accidents. The discussion on P12, l22-23 may partlly explain this in that it is indicated that those kids with SDH and RH often lacked brain edema or contusion.
Kids with simple falls often have small subjacent contact SDHs, but lack accompanying brain injury and could have a few minor/non-specific RH. Unfortumately, the data to decide whether this is the explanation is lacking as RH were binomally present or absent and more concerning SDHs were not separated form simple subjacent contact injuries. This confo\ict with reality will need more elaboration in the discussion.

**Author’s Response:** The following sentences were added.

Change to text: Page 13, Lines 14-16

Furthermore, since we did not assess the severity of subdural hematomas or retinal hemorrhages in this study, it is possible that these factors were also milder in this group of patients.

6) Also, adding Table 5 helps a little to reassure readers that those minor injury mechanisms were seen at all participting institutions, as the authors noted. But the did not comment that hospital A saw 6.7% were abusive and hospital C 66.7% abusive, but the other 4 found no abuse cases among these minor trauma events. This reinforces my original opinion that although Japanese medicine is doing better at recognizing that abuse occurs, that remains very spotty. And at some institutions, fabricated history is usually accepted at face vale, while some are doing better. Those %s of abuse diagnosis need to be addressed in the discussion ( in the vicinity of page 12, line 20-24) and comments are needed about the real possibility that abuse ifs still frequently remaining undiagnosed.

**Author’s Response:** The following sentences were added.

Change to text: Page 13, Lines 17-19

The different percentages of patients identified as abusive in different facilities may be due to regional differences in abuse recognition.

7) Page 10, L10: It is interesting that the minor injury event kids had had a lot of skull Fxs, but no EDHs or brain contusions. Both are commonly present with primarily impact events that cause skull Fxs at the same time.

**Author’s Response:** It is true that there were many cases with only skull fractures and no intracranial abnormalities. However, since this is not the focus of the paper, we will not discuss about this in the text.

8) Somewhere in Results I'd like to have reported. how many abused vs non-abused kids had SDH + RH vs SDH - RH and RH _ SDH vs RH - SDH.

**Author’s Response:** We have created a new Table.

Change to text: Page 11, Lines 10-13 + Table 6 is added.

The relationship between subdural hematoma, retinal hemorrhage and nonaccidental/accidental is shown in Table ６.

9) P 13, L5; I think it is important to recognize that Japanese children are highly unlikely to be uniquely susceptible to SDHs from minor falls. Obviously this would be racially stereotyping, when race is a poor proxy for anything biologic.

If it were true, we would expect Japanese children to experience the same risk if they lived elsewhere, say the US where they sustain fewer injuries than others.

We also would expect the same brain injury susceptabilty to extend to other cause of TBI, say falls from significant heights or MVAs, but the current data don't support that.

**Author’s Response:** We consider that differences by race and culture cannot be ignored. It is also true that the cranial shape of infants differs between Westerners and Japanese, and that head size differs by race, especially for Japanese breast-fed infants, who are larger than the global standard. As you know, macrocephaly is prone to subdural hematoma.

We are also concerned that Japanese or Asian cases of subdural hematoma combined with retinal hemorrhage in countries other than Japan, especially in the U.S, may not be reported in the scientific journal because medical institutions and /or public agencies may automatically diagnose the cause of the injury as abuse. Gardner's paper which we removed from citation according to the reviewer's suggestion, was about Asian infant.

Change to text: Page 13, Lines 21-24, Page 14, Line 1

In fact, the cranial shape of infants differs between Westerners and Japanese {Koizumi, 2010 #179}. In addition, head size varies by race {Natale, 2014 #182} and in particular, Japanese breast-fed infants have larger head circumference than the world standard. {Tanaka, 2013 #181}　 We assume that the size and shape of the cranium may change the impact of head trauma.

10) I expect this is a hold over form the era when it was culturally unacceptable to admit that abuse occurred in Japan (all Aoki's early writing). I think these are ideas that have long over-lived their time and should be buried by more critical case reviews.

**Author’s Response:** We believe that we need to fully examine one idea before burying it. The following sentences were added.

Change to text: Page 14, Lines 2-3

Future research is needed to determine whether “self-inflicted falls” in this study were in fact caused by abuse or by accidental occipital impact.

11) It would be worth adding that these is at least a case series to suggest falls with occipital impacts may be more morbid than with other other impact sites. Atkinson Pediatric Emerg care 2018;34:837.

**Author’s Response:** The following sentences were added.

Change to text: Page 13, Lines 3-5

This report, and our findings may suggest that falls due to impact on the occipital region may be more morbid than other impact sites.

P 15, L7: I appreciate the addition of the discussion the NAT may be underestimated. The following statements could be edited to more clearly state that no dividing RH into type number, location, severity in some way precludes conclusions anbout minor vs more specific complex, profuse RH patterns with or without schisis.

**Author’s Response:** The following sentences were added.

Change to text: Page 15, Lines 11-13

Whether retinal hemorrhage was unilateral or bilateral, association with retinal schisis, or the severity of the hemorrhage was not considered.

I think it would be OK in the conclusion to add that brain edema in multi-variable analysis was also associated with RH (see Binenbaum Pediatrics. 2013;132:e430) . And a sentence saying that the association of RH with SDH & brain edema can't imply the SDH caused the RH vs the same forces that caused the SDH, brain edema.

**Author’s Response:** Thank you for your advice. However, we did not make any changes because we thought the current conclusion was simpler and better.
